# Effectiveness of Opioids for Low Back Pain: A Systematic Review of Randomised Controlled Trials

**DOI:** 10.7759/cureus.110683

**Published:** 2026-06-11

**Authors:** Arathy Krishna Suresh Babu

**Affiliations:** 1 General Medicine, Ulster University, Birmingham, GBR; 2 Medicine, JSS Medical College, Mysore, IND

**Keywords:** buprenorphine, chronic opioid use, low back pain (lbp), opioid analgesics, opioid side effects, opioid use, oxycodone, pain relief, randomised controlled trials (rcts), systematic review

## Abstract

Chronic low back pain (LBP) is a major cause of disability around the world, and pharmaceutical treatments like opioids are often used to treat it. However, the efficacy and safety of opioids in LBP continue to be controversial due to inconsistent results and associated side effects. This systematic review assesses the effectiveness and safety of opioid medication in the treatment of LBP. A systematic search was performed utilising various electronic databases, including PubMed, Scopus, Google Scholar, MEDLINE, the Cochrane Library, and Web of Science, following a structured methodology in accordance with Preferred Reporting Items for Systematic Reviews and Meta-Analyses (PRISMA) principles. Randomised controlled trials that were published in English within the past two decades and evaluated the use of opioids in adults with LBP were included. The Cochrane Risk of Bias tool and the Critical Appraisal Skills Programme (CASP) checklist were used to screen for bias, and a narrative synthesis was done because the studies were not all the same. There were six randomised, double-blind, placebo-controlled trials involving between 83 and 905 people. Opioid formulations, including buprenorphine, hydrocodone, oxycodone/naloxone, and oxymorphone, exhibited overall efficacy in diminishing pain intensity relative to placebo, especially in opioid-naïve individuals. But the magnitude and consistency of the advantage varied across studies, and some treatments showed only a small statistical difference. There were only a few reports of functional improvement, and they were not always constant. Nausea, constipation, and drowsiness were common side effects, although long-term safety outcomes were not fully evaluated. In general, opioids may help with short-term pain relief in people with LBP, but they aren't very useful in the long term because they aren't very effective and very safe and there isn't much evidence for their long-term use. It is advisable to select patients and use them with caution. More high-quality research is needed to understand their role in long-term management better.

## Introduction and background

Low back pain (LBP) is a highly prevalent musculoskeletal condition affecting individuals across all age groups and remains one of the leading causes of disability, work absenteeism, and years lived with disability worldwide, representing a significant global health burden. Patients typically present with pain localised to the lumbar region, which may radiate to the buttocks or lower limbs, often resulting in significant functional limitation and reduced quality of life. Although LBP is common, its underlying aetiology is often multifactorial and not always clearly identifiable. It may result from conditions such as muscle strain, osteoarthritis, ligament sprain, intervertebral disc herniation, and sciatica, while risk factors such as poor posture, sedentary lifestyle, obesity, advancing age, and occupational strain may further increase susceptibility.

The management of LBP is multifaceted and includes non-pharmacological, pharmacological, and surgical approaches. Conservative strategies such as exercise therapy, behavioural interventions, and physical rehabilitation are widely recommended, although pharmacological management remains commonly used in clinical practice [1-3]. Opioids continue to be prescribed for selected patients with chronic LBP, particularly in North America, despite increasing concerns regarding long-term efficacy and safety [4]. While opioids may provide short-term analgesic benefit, their impact on functional outcomes remains limited, with evidence suggesting only modest improvements compared with placebo [2,4].

Opioid use is associated with substantial risks, including tolerance, opioid-induced hyperalgesia, dependence, and overdose-related mortality [4]. Common adverse effects such as nausea, constipation, and sedation are frequently reported, while long-term use may also be associated with depression and sexual dysfunction. Increasing trends in long-term opioid prescribing for chronic non-cancer pain have raised further concerns regarding the safety and appropriateness of use [5]. Overall, evidence regarding the effectiveness of opioids in LBP remains inconclusive due to methodological limitations, short follow-up periods, and limited generalisability of existing studies [6,7]. Important uncertainties also remain regarding optimal dosing strategies, long-term outcomes, and the overall risk-benefit balance, highlighting a clear evidence gap.

LBP arises from a complex interplay of anatomical and physiological factors. Pain may originate from intervertebral disc degeneration, facet joint pathology, sacroiliac joint dysfunction, nerve root involvement, or conditions such as spinal stenosis and spondylolisthesis [8-10]. Other contributors include myofascial pain, peripheral nerve disorders, and central sensitisation mechanisms, which further complicate diagnosis and management [11]. Given this complexity, treatment should be individualised according to the patient's clinical presentation and underlying pathology, incorporating appropriate combinations of conservative, pharmacological, and, when necessary, surgical options.

Opioids are a class of analgesic agents used in the management of moderate to severe pain and exert their effects by acting on μ (mu), δ (delta), and κ (kappa) opioid receptors within the central and peripheral nervous systems [12]. Although effective in acute and cancer-related pain, their role in chronic LBP remains controversial. Current clinical recommendations suggest that opioids should be reserved for patients who have not responded adequately to first-line pharmacological and non-pharmacological interventions, due to their associated risks and limited evidence of long-term benefit [13]. While opioids may provide short-term pain relief, their overall clinical utility requires careful consideration given concerns regarding safety, dependence, and variable treatment response.

Therefore, the objective of this systematic review is to evaluate the effectiveness and safety of opioid therapy in chronic LBP in adults compared with placebo or non-opioid treatments, with particular focus on pain relief, functional outcomes, and adverse effects.

## Review

Methodology

Study Design

This study was conducted as a systematic review to evaluate the effectiveness and safety of opioids in the treatment of LBP. A systematic approach was used to identify, appraise, and synthesise relevant evidence, aiming to minimise bias and provide a comprehensive and objective assessment. A meta-analysis was considered; however, it was not performed due to heterogeneity among the included studies. Heterogeneity arose from differences in opioid formulations, dosing regimens, treatment duration, outcome measures, patient characteristics, and follow-up periods across the included studies. 

Statistical Analysis

As this systematic review did not include a meta-analysis, no formal statistical pooling of effect sizes was performed. Consequently, no fixed-effects or random-effects models were applied. Heterogeneity was assessed qualitatively based on variations in study design, populations, interventions, and outcome measures rather than statistically (e.g., I² statistics). Publication bias was not formally evaluated due to the absence of meta-analytic pooling. Individual study-level statistical results, including p-values reported in the original studies, were extracted and presented descriptively.

Search Strategy

A comprehensive literature search was conducted in 2023 using electronic databases, including PubMed, Scopus, and Google Scholar. Additional databases, such as the Cochrane Library, MEDLINE, and Web of Science, were also searched to ensure broad coverage of the relevant literature. Studies published over the preceding 20 years (2003-2023) were eligible for inclusion to capture the available evidence at the time of the search.

Search strategies were tailored to each database using database-specific controlled vocabulary (e.g., Medical Subject Headings (MeSH) terms in PubMed) and free-text keywords combined with Boolean operators (AND, OR) in order to maximise sensitivity and specificity.

Table 1 presents the search strategy using relevant keywords. A combination of MeSH terms and keywords was used with Boolean operators (AND, OR). The search terms included the following: "effectiveness", "efficacy", "outcome", "treatment success", "pain relief", "opioids", "opioid analgesics", "narcotics", "morphine", "codeine", "oxycodone", "fentanyl", "hydrocodone", "low back pain", "lower back pain", "lumbar pain", and "backache". 

**Table 1:** Search strategy: keywords

Search engines	Boolean search query	Total records
PubMed	(“Effectiveness” OR “efficacy” OR “outcome” OR “treatment success” OR “pain relief”) AND (“opioids” OR “opioid analgesics” OR “narcotics” OR “morphine” OR “codeine” OR “oxycodone” OR “fentanyl” OR “hydrocodone”) AND (“low back pain” OR “lower back pain” OR “lumbar pain” OR “backache”)	1133
Google Scholar	(“Effectiveness” OR “efficacy” OR “outcome” OR “treatment success” OR “pain relief”) AND (“opioids” OR “opioid analgesics” OR “narcotics” OR “morphine” OR “codeine” OR “oxycodone” OR “fentanyl” OR “hydrocodone”) AND (“low back pain” OR “lower back pain” OR “lumbar pain” OR “backache”)	46
Scopus	(“Effectiveness” OR “efficacy” OR “outcome” OR “treatment success” OR “pain relief”) AND (“opioids” OR “opioid analgesics” OR “narcotics” OR “morphine” OR “codeine” OR “oxycodone” OR “fentanyl” OR “hydrocodone”) AND (“low back pain” OR “lower back pain” OR “lumbar pain” OR “backache”)	90

Study Selection

Studies were selected through a structured, multi-stage screening process in line with Preferred Reporting Items for Systematic Reviews and Meta-Analyses (PRISMA) guidelines. Electronic searches were conducted in PubMed, Google Scholar, Web of Science, Scopus, and the Cochrane Library, alongside manual searches of reference lists to identify additional relevant studies. Screening was carried out in three stages: title screening to remove irrelevant records, abstract screening to assess relevance, and full-text review to determine eligibility based on predefined inclusion and exclusion criteria. Any disagreements were resolved through discussion and consensus.

Eligibility Criteria (PICO Framework)

The PICO framework was used to establish the eligibility criteria for this systematic review, enabling studies to be selected in a structured and systematic manner. The population (P) comprised adults experiencing LBP. The intervention (I) involved opioid medications, including morphine, codeine, oxycodone, fentanyl, and hydrocodone, while the comparator (C) included placebo, non-opioid pharmacological treatments, or standard care interventions as appropriate. The outcomes (O) of interest were pain relief, functional improvement, quality of life, and adverse effects. Inclusion criteria included randomised controlled trials published in English within the past 20 years that investigated the efficacy of opioids in LBP populations [14]. Exclusion criteria included case reports, review articles, studies without full-text availability, unpublished online reports, and abstracts lacking sufficient data, as these were considered to provide insufficient primary or methodologically robust evidence [15]. Overall, these criteria ensured that the review included high-quality, relevant, and methodologically sound studies suitable for analysis.

Table 2 shows the rules for choosing which articles to include and which to leave out of this study. It emphasises a focus on current, English-language randomised controlled trials evaluating opioid efficacy in LBP while omitting irrelevant, low-quality, or incomplete findings.

**Table 2:** Inclusion and exclusion criteria

Inclusion criteria	Exclusion criteria
Articles published in the last 20 years	Unpublished papers uploaded online
Articles in the English language	Articles with only abstracts and no full text
Articles that estimated the effectiveness of opioids	Study reviews
Studies with participants experiencing low back pain	Case studies
Randomised controlled trials	Articles that were not in the English language
Articles with relevant outcomes	Review articles

Outcome Measures

The primary outcomes assessed included pain intensity and functional improvement. Secondary outcomes included quality of life, adverse effects, patient-reported outcomes, long-term effects, mental health outcomes, and return to work.

Risk of Bias Assessment

The Cochrane Risk of Bias tool and the Critical Appraisal Skills Programme (CASP) checklist were used to assess bias and ensure that the study quality was thoroughly evaluated. The main areas observed were selection bias (random sequence generation and allocation concealment), performance bias (blinding of participants and staff), detection bias (blinding of outcome assessment), attrition bias (incomplete outcome data), and reporting bias (selective outcome reporting). Factors specifically relevant to opioid research were further examined, including dosage variability, treatment duration, participant adherence, and the documentation of adverse effects. The CASP criteria were used to examine further each study's validity, reliability, and usefulness. In general, the included studies showed a moderate risk of bias, primarily due to insufficient blinding, limited follow-up, and differences in outcome measures [16,17].

Table 3 presents the risk of bias assessment of the included randomised controlled trials using Cochrane domains. Most studies showed a low to moderate risk of bias, with some concerns related to incomplete outcome data and allocation concealment.

**Table 3:** Risk of bias table

Study	Random sequence generation	Allocation concealment	Blinding (participants and personnel)	Blinding (outcome assessment)	Incomplete outcome data	Selective reporting	Overall risk
Rauck et al. (2016) [18]	Low risk	Low risk	Low risk	Low risk	Moderate risk	Low risk	Low-moderate
Hale et al. (2015) [19]	Low risk	Low risk	Low risk	Low risk	Moderate risk	Low risk	Low-moderate
Wen et al. (2015) [20]	Low risk	Low risk	Low risk	Low risk	Low risk	Low risk	Low
Cloutier et al. (2013) [21]	Low risk	Unclear risk	Low risk	Low risk	Moderate risk	Low risk	Moderate
Steiner et al. (2011) [22]	Low risk	Low risk	Low risk	Low risk	Low risk	Low risk	Low
Hale et al. (2007) [23]	Low risk	Unclear risk	Low risk	Low risk	Moderate risk	Low risk	Moderate

Benefit vs. Risk Analysis

A benefit-risk analysis was performed to assess the balance between the therapeutic benefits and possible adverse effects of opioid utilisation in LBP. The evaluated advantages included pain alleviation and enhancement of functional outcomes, whereas the risks concentrated on undesirable effects, including nausea, sedation, constipation, and the possibility of dependency and misuse. The research results included were carefully examined to determine how useful opioids are in general. This approach facilitated a balanced and structured interpretation of the evidence to support informed clinical decision-making [17].

Data Extraction and Synthesis

Data were extracted using a predefined standardised data extraction form developed prior to study selection. The extraction process included study design, sample size, participant characteristics, intervention details, comparator groups, outcome measures, and key findings. Data extraction was performed independently and cross-checked for accuracy to reduce error and bias. Any disagreements between reviewers were resolved through discussion and consensus.

Given the substantial heterogeneity across studies in terms of design, populations, opioid formulations, dosing regimens, outcome measures, and follow-up durations, statistical pooling of results was not appropriate. Therefore, a meta-analysis was not conducted.

No quantitative synthesis measures such as pooled effect sizes, 95% confidence intervals, I² statistics, or publication bias assessments were performed due to methodological heterogeneity and variation in outcome reporting across studies.

Instead, a structured narrative synthesis approach was used to systematically compare and interpret findings across studies, focusing on pain reduction, functional outcomes, and adverse effects.

As the review utilised previously published data, ethical approval was not required.

Results

Literature Search

The literature search was conducted in three steps to ensure that only reliable and relevant research was selected. At first, titles were filtered using keywords to identify studies related to the research issue. Next came abstract screening, which reviewed the goals, methods, and main results of the selected studies and excluded those that weren't relevant. Lastly, full-text screening was conducted to determine whether the studies met the inclusion criteria based on their design, methods, findings, and overall relevance. Studies that did not match these criteria were not included. This three-stage screening approach ensured that only high-quality papers closely related to the study's issue were included.

PRISMA Flow Diagram

**Figure 1:**
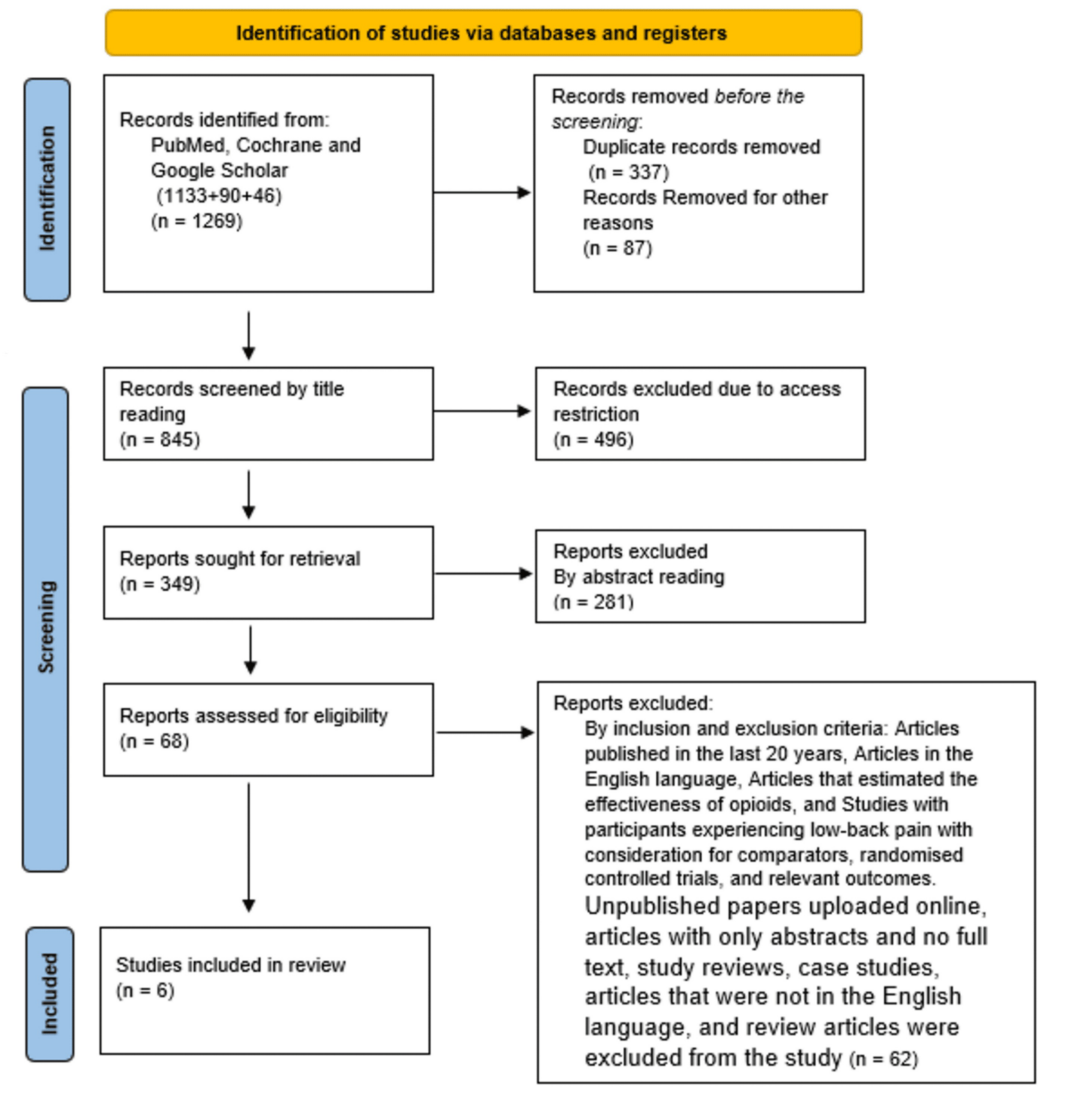
PRISMA chart PRISMA: Preferred Reporting Items for Systematic Reviews and Meta-Analyses

Figure 1 shows the PRISMA flow diagram which illustrates the study selection process. Three databases, namely, PubMed (1133 records), Cochrane (90 records), and Google Scholar (46 records), initially found 1269 records. After removing 337 duplicate records and 87 records not used for other initial reasons, 845 studies remained for title review. At this point, 496 records were left out, mostly because they couldn't be accessed or the full texts weren't available. The remaining 349 papers were screened for abstracts, and 281 were discarded because they didn't address the research question, had the wrong primary focus, or used inappropriate study designs. A total of 68 full-text articles were then assessed for eligibility. Of these, 62 reports were excluded according to predetermined inclusion and exclusion criteria, which included studies published beyond the last 20 years, non-English-language articles, studies that did not assess opioid efficacy in LBP, non-randomised designs, and reports that did not provide full-text access, including abstracts, case studies, and review articles. After this thorough screening process, six studies passed all the requirements and were included in the final systematic review.

Evidence Synthesis

The systematic review of the six trials indicates that opioids may offer effective short-term analgesia for LBP therapy. However, the extent of benefit differs among formulations and demographics. In opioid-naïve individuals suffering from chronic LBP, both transdermal and buccal buprenorphine, together with once-daily hydrocodone, were consistently linked to substantial pain alleviation and satisfactory tolerability [18-22]. Extended‑release hydrocodone and controlled‑release oxycodone/naloxone showed inconsistent findings, with some studies reporting pain reduction but the results not reaching statistical significance across primary outcomes (p=0.0527; p=0.0862) [19,21]. In contrast, oxymorphone extended-release demonstrated considerable efficacy in opioid-experienced patients, suggesting its potential utility for persons unresponsive to other opioid therapy [18]. Individual trial results further support these findings. For example, buccal buprenorphine (p=0.0012) [18], hydrocodone formulations (p=0.032; p=0.0016) [19,20], and buprenorphine transdermal systems (p=0.0104) [22] all showed significant pain relief. Oxycodone/naloxone (p=0.0527; p=0.0862) [21] only showed marginal statistical significance, and oxymorphone extended-release (p<0.001) [23] showed strong efficacy in patients who had already used opioids. In general, these results suggest that opioids may help with short-term pain relief in chronic LBP, but the fact that their effectiveness varies means that we should be careful about how we interpret them.

Table 4 presents the main features and results of the included randomised controlled trials. It shows that most opioid treatments worked better than a placebo, but the size and consistency of the benefit varied from study to study.

**Table 4:** Evidence table LBP: low back pain; MMRM: mixed-effects model repeated measures; BBUP: buccal buprenorphine; ER: extended-release; CR: controlled-release; VAS: Visual Analogue Scale; BTDS: buprenorphine transdermal system; OPANA ER: oxymorphone extended-release

Sl. no.	Author/year	Study design	Participants	Sample size	Comparator	Period	Outcome measure	Result	Statistical analysis
1	Rauck et al. (2016) [18]	Randomised double-blind placebo-controlled clinical trial	Opioid-naïve adults with moderate to severe chronic LBP	749	Placebo	12 weeks	Pain reduction	Opioid (BBUP) treatment provided better pain relief and fewer side effects than a placebo	p=0.0012 (MMRM test)
2	Hale et al. (2015) [19]	Randomised double-blind placebo-controlled clinical trial	Patients with osteoarthritis or lower back pain	294	Placebo	12 weeks	Pain relief	Higher doses of opioids (hydrocodone ER) showed better pain relief than placebo	p=0.032 (Wilcoxon rank-sum test)
3	Wen et al. (2015) [20]	Randomised double-blind placebo-controlled clinical trial	Patients with moderate to severe chronic LBP	905	Placebo	12 weeks	Pain score reduction	Opioid (hydrocodone) treatment significantly reduced pain scores compared to placebo	p=0.0016 (MMRM test)
4	Cloutier et al. (2013) [21]	Randomised double-blind placebo-controlled clinical trial	Patients with chronic LBP	83	Placebo	8 weeks	Pain intensity	Opioid treatment (oxycodone/naloxone CR) significantly reduced pain compared to placebo	VAS: p=0.0527; ordinal scale: p=0.0862
5	Steiner et al. (2011) [22]	Randomised double-blind placebo-controlled clinical trial	Opioid-naïve patients with chronic LBP	541	Placebo	12 weeks	Pain improvement	Treatment with opioids (BTDS) resulted in a more significant reduction in pain scores compared to placebo	p=0.0104 (hybrid imputation method)
6	Hale et al. (2007) [23]	Randomised double-blind placebo-controlled clinical trial	Opioid-experienced patients	392	Placebo	12 weeks	Pain intensity	Opioid (OPANA ER) treatment significantly reduced pain compared to placebo	p<0.0001 (Kaplan-Meier survival method, log-rank test, rank-sum procedure)

Findings of the Included Studies

The studies were randomised, double-blind, placebo-controlled trials assessing the efficacy and safety of opioids in persons with persistent LBP, including both opioid-naïve and opioid-experienced cohorts. The number of participants in each study ranged from 83 to 905, and the studies lasted 8-12 weeks. All of them employed a placebo as the control. The study looked at many types of opioids, such as buprenorphine, hydrocodone, oxycodone/naloxone, and oxymorphone. In general, most trials showed that opioids helped with short-term pain relief, but the amount and consistency of the benefit varied. Buccal buprenorphine showed significant decreases in pain intensity with favourable tolerability in opioid-naïve patients [18], whereas hydrocodone formulations displayed improved analgesia, particularly at elevated doses, with generally acceptable safety profiles [19,20]. Controlled-release oxycodone/naloxone resulted in slight pain relief that was almost statistically significant [21]. In contrast, the buprenorphine transdermal system enhanced pain management, increased sleep quality, and decreased the necessity for supplementary analgesics, accompanied by anticipated opioid-related side effects [22]. In patients with prior opioid exposure, oxymorphone extended-release had substantial analgesic effects and enhanced patient satisfaction [23].

Table 5 summarises the main characteristics and conclusions of this research. It shows that while opioids can help with pain in the short term, their effectiveness and statistical significance vary between formulations and patient groups.

**Table 5:** Findings table LBP: low back pain; ER: extended-release; CR: controlled-release; OPANA ER: oxymorphone extended-release; AEs: adverse events

Study	Population	Intervention	Key findings	Safety/tolerability	Conclusion
Rauck et al. (2016) [18]	Opioid-naïve patients with moderate to severe chronic LBP	Buccal buprenorphine	Effective pain relief and better responder rates than placebo	Well-tolerated; fewer opioid-related adverse effects; some discontinuations	Effective and well-tolerated for opioid-naïve chronic LBP
Hale et al. (2015) [19]	Patients with osteoarthritis or LBP	Hydrocodone ER	Improved pain relief at higher doses (post hoc); primary endpoint not significant	Generally well-tolerated; common adverse effects: constipation, nausea	Effective at adequate doses
Wen et al. (2015) [20]	Moderate to severe chronic LBP patients	Once-daily hydrocodone	Significant reduction in pain scores vs. placebo	No major safety concerns; well-tolerated	Effective and safe for chronic LBP
Cloutier et al. (2013) [21]	Chronic LBP patients	Oxycodone/naloxone CR	Pain reduction observed; marginal statistical significance	Comparable adverse effects to placebo; acceptable safety profile	Potential benefit but less definitive
Steiner et al. (2011) [22]	Opioid-naïve chronic LBP patients	Buprenorphine transdermal system	Significant pain reduction, improved sleep, reduced analgesic use	Typical opioid adverse effects; no unexpected safety issues	Effective and well-tolerated
Hale et al. (2007) [23]	Opioid-experienced chronic LBP patients	OPANA ER	Significant pain reduction; improved satisfaction; lower discontinuation	Tolerable with expected opioid AEs	Effective option for opioid-experienced patients

Critical Appraisal/Risk of Bias

The CASP checklist for randomised controlled trials was used to rate the methodological quality of the included studies. In general, the studies were methodologically valid because they were all randomised, double-blind, placebo-controlled trials with specific study questions. Most studies indicated similar baseline characteristics across groups and thorough documentation of intervention effects. However, certain problems were identified, including participant dropout, incomplete follow-up in some studies, outcomes that were only marginally statistically significant, and possible limitations in generalisability due to rigorous inclusion criteria or enhanced study designs. Even with these problems, the total body of data was thought to be of moderate quality and gave helpful information about how well and safely opioids work for persistent LBP.

Discussion

The findings of this systematic review indicate that opioids offer effective short-term analgesia for patients with chronic LBP, especially in opioid-naïve cohorts. The randomised controlled trials consistently indicated that opioid formulations, including buprenorphine, hydrocodone, and oxymorphone, significantly reduced pain intensity in comparison to placebo [18-23]. These results align with previous systematic reviews demonstrating that opioids provide modest short-term analgesic benefits but limited functional improvement in chronic LBP [6,7]. However, the magnitude of benefit varied across studies, with some trials reporting only small or no significant effects. This highlights variability in treatment effectiveness across studies.

A significant finding of this review is the difference in short-term efficacy among various opioid formulations and patient populations. Buprenorphine-based therapies and hydrocodone had consistent advantages in opioid-naïve patients, evidenced by enhanced pain scores and responder rates [18-20,22]. Controlled-release oxycodone naloxone, on the other hand, demonstrated questionable statistical significance, indicating less consistent efficacy [21]. Furthermore, oxymorphone extended-release proved efficacious in patients with prior opioid exposure, suggesting that previous opioid use may affect treatment response [23]. Similar variability in treatment response has been reported in previous studies, highlighting differences in efficacy based on opioid formulations and patient characteristics [7]. This variation in efficacy across opioid formulations and patient populations has also been reported in previous systematic reviews, suggesting that treatment response may depend on both drug characteristics and patient factors [6,7]. These results highlight the importance of personalised treatment strategies, necessitating that opioid selection be customised according to patient characteristics and previous treatment experiences.

Despite these short-term benefits, the evidence for long-term functional outcomes remains limited. Although pain scores improved in many studies, further improvements in functional outcomes were less apparent [18-24]. Clinical recommendations confirm this, suggesting that opioids may not lead to meaningful functional improvement even when pain relief is achieved [25]. Additionally, most included trials were of short duration, which limits conclusions regarding sustained efficacy and long-term functional benefit.

Safety remains a significant concern. Studies consistently found common short-term side effects, including nausea, constipation, dizziness, and drowsiness [18-22]. These adverse effects were generally manageable but led to treatment discontinuation in some patients. This is in line with other studies showing that opioids are associated with more adverse events than non-opioid treatments. This is supported by large systematic reviews and clinical guidelines, which highlight increased risks of adverse events, dependence, and misuse associated with opioid therapy [5]. These findings are consistent with previous evidence demonstrating higher rates of adverse events and discontinuation among patients receiving opioid therapy compared to non-opioid treatments [6]. Additionally, while the included trials primarily report short-term safety outcomes, prolonged opioid use is associated with significant risks, including dependence, misuse, and overdose [5,6,24], which must be considered when evaluating short-term benefits.

Heterogeneity among the included studies also limits the strength of the conclusions. Differences in study design, patient groups, types of opioids, dose schedules, and outcome measures led to findings that were not always consistent. This heterogeneity precluded a quantitative meta-analysis and necessitated a narrative synthesis, potentially constraining comparisons between trials.

The merits of this study include the inclusion of high-quality evidence, with only randomised, double-blind, placebo-controlled trials considered, alongside a robust and systematic methodological approach guided by PRISMA, CASP, and Cochrane frameworks. However, it is essential to recognise the limitations. The limited number of trials (n=6) constrains the generalisability of findings, and the emphasis on short-term outcomes restricts the understanding of long-term efficacy and safety. Moreover, potential publication bias and selective reporting may have impacted the overall results.

Limitations

When analysing the results of this systematic review, it is important to consider several limitations. The relatively small number of included studies (n=6) may limit the generalisability of the findings, despite all being randomised, double-blind, placebo-controlled trials. Additionally, most studies focused on short-term outcomes (8-12 weeks), restricting the assessment of long-term efficacy and safety, including risks such as dependence, tolerance, and misuse. There was also notable heterogeneity across studies in terms of design, patient populations (opioid-naïve versus opioid-experienced), opioid formulations, dosing regimens, and outcome measures, which contributed to variability in findings and made quantitative meta-analysis unfeasible, necessitating a narrative synthesis approach [18-23]. Additionally, inconsistencies in outcome definitions and reporting may have influenced interpretation. The inclusion of only English-language, published studies introduces potential publication and selection bias, as positive findings are more likely to be reported. Some trials also demonstrated marginal or non-significant results [19,21], further affecting consistency. Furthermore, limitations within individual studies, such as participant attrition, inadequate follow-up, and restrictive inclusion criteria, may have impacted the overall reliability of the findings.

Future research

Future research should focus on evaluating the long-term safety and efficacy of opioids in larger and more diverse patient populations, with the use of standardised outcome measures to improve comparability across studies. Additionally, further studies are needed to assess the risks of dependence, tolerance, and misuse, as well as their impact on functional outcomes and quality of life.

## Conclusions

Chronic LBP is a leading cause of disability worldwide, and opioids are commonly prescribed for its management. This systematic review of six randomised controlled trials indicates that opioids can provide modest short-term analgesic benefit in persistent LBP. Most studies showed that opioids reduced pain intensity significantly compared to a placebo. Buprenorphine and hydrocodone formulations had the most reliable efficacy, especially in opioid-naïve patients, whereas oxycodone/naloxone displayed variable efficacy, and oxymorphone extended-release proved useful in opioid-experienced persons. Although opioids were generally tolerated, they often reported common side effects. It is important to note that improvements in functional outcomes were limited and inconsistent. Additionally, the current evidence is limited to short-term follow-up, and there is insufficient data on long-term safety and risk factors, such as dependence and misuse. In general, opioids may help some people with short-term pain relief, but their effectiveness varies, their functional benefits are limited, and they are associated with significant risks. These findings support cautious, individualised use rather than routine use in the management of chronic LBP.
